# Coassembly of Complementary Polyhedral Metal–Organic Framework Particles into Binary Ordered Superstructures

**DOI:** 10.1021/jacs.4c07194

**Published:** 2024-07-26

**Authors:** Lingxin Meng, Javier Fonseca, Roberto Sánchez-Naya, Amir Mohammad Ghadiri, Inhar Imaz, Daniel Maspoch

**Affiliations:** † Catalan Institute of Nanoscience and Nanotechnology (ICN2), CSIC, and Barcelona Institute of Science and Technology Campus UAB, 08193 Bellaterra, Barcelona, Spain; ‡ Departament de Química, Facultat de Ciències, Universitat Autònoma de Barcelona, 08193 Bellaterra, Spain; § ICREA, Pg. Lluís Companys 23, 08010 Barcelona, Spain

## Abstract

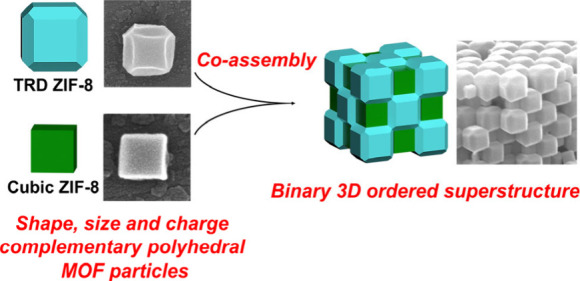

Here we report the formation of a 3D NaCl-type binary porous superstructure *via* coassembly of two colloidal polyhedral metal–organic framework (MOF) particles having complementary sizes, shapes, and charges. We employed a polymeric-attenuated Coulombic self-assembly approach, which also facilitated the coassembly of these MOF particles with spherical polystyrene particles to form 2D binary superstructures. Our results pave the way for using MOFs to create sophisticated superstructures comprising particles of various sizes, shapes, porosities, and chemical compositions.

Self-assembly of colloidal particles into ordered three-dimensional (3D) superstructures offers novel opportunities for producing functional materials with photonic, magnetic, electronic, catalytic, mechanical, and thermal properties.^1,2^ Traditionally, isotropic particles such as polymers^2−6^ (e.g., polystyrene [PS]), silica,^7^ and more recently, covalent organic frameworks (COFs)^8^ have been utilized to fabricate ordered superstructures that typically exhibit face-centered cubic (fcc) or hexagonal close-packed (hcp) lattices. Recently, anisotropic polyhedral particles such as those of gold,^9−12^ silver,^13^ quantum dots,^14,15^ metal–organic frameworks (MOFs),^16−21^ or colloidal clusters composed of isotropic particles^22^ also have been employed as colloidal building units. Due to their varied polyhedral shapes, such particles can arrange themselves into multiple lattices, including Kelvin,^13^ Minkowski,^13,17^ diamond,^22^ and others,^9,10,23^ which exhibit greater packing fractions as well as diverse geometric features.^1,2,9−13,15,16,23,24^

Another strategy for accessing different superstructures involves the simultaneous assembly of two types of colloidal particles.^3,5,6,25−30^ Although more challenging than self-assembly of the same particles, the coassembly of two different particles enables the formation of ordered binary superstructures whose packing resembles the structures formed by two atoms; in other words, each particle acts as an atom.^3,5,6,22,26,30−33^ A clear example is NaCl, whose structure could be replicated by the coassembly of two types of spherical particles (e.g., silica, poly(methyl methacrylate), *etc.*).^3,5,6,26,30,31^ This approach not only expands the accessible range of ordered superstructures that exhibit the aforementioned properties but also aids in understanding and simulating the formation of atomic and molecular crystals.^34^ Additionally, this approach may eventually help researchers to discover unprecedented unnatural metamaterials and exotic superlattices.^13^

To date, far fewer 3D binary superstructures have been reported compared to those assembled from a single particle type. Literature examples of the former include a few 3D binary superstructures assembled from two different spherical particles^3,5,6,25−30,32,33^ or from one spherical and one polyhedral particle.^22,31,35−39^ However, the assembly of 3D superstructures from two different polyhedral particles has only been described in two studies.^40,41^ This scarcity is understandable when considering the complexity of ordering two different polyhedral particles along three dimensions, as it requires the two particle types to be complementary in both size and shape while also demanding control over the interactions that force their pairing assembly. Nevertheless, being able to control this ordering is crucial for the assembly of new and desired lattices, as the aforementioned complementarity can dictate the final symmetry of the superstructure. In 2015, Mirkin et al. first showcased control over this assembly by employing convex and concave cubic nanoparticles, which were complementary in both size and shape, to form a 3D superstructure *via* pairing of complementary DNA interactions.^41^ More recently, the same group extended the use of such interactions to assemble 10 new 3D superstructures from other pairs of polyhedral nanoparticles (e.g., cuboctahedral + octahedral nanoparticles; tetrahedral + octahedral nanoparticles; *etc.*).^40^

Herein we report the use of colloidal polyhedral MOF particles having complementary sizes, shapes, and charges to create 3D binary porous superstructures. Specifically, we have demonstrated the formation of an fcc, NaCl-type porous superstructure *via* coassembly of oppositely charged zeolitic imidazolate framework-8 (ZIF-8) particles of two topologies: truncated rhombic dodecahedral (TRD), and cubic (C). To balance repulsion and Coulombic attraction between the particles, we employed a polymeric-attenuated Coulombic self-assembly (PACS) approach based on Pluronic F127 and NaCl.^6^ Pluronic F127 initially forms a uniform brush on the ZIF-8 particle surface, controlling steric repulsion,^42^ whereas NaCl modulates the electrostatic attractions between the oppositely charged ZIF-8 particles. Furthermore, the use of Pluronic F127 as a long polymer surfactant ligand that is coated onto polyhedral particles does not compromise particle anisotropy or affect shape-directed phenomena.^37,40,43,44^

Prior to assembling our target superstructure, we first optimized the electrostatic forces and the PACS strategy to facilitate the pairing of the polyhedral ZIF-8 particles. To this end, we initially tested this approach in the binary assembly of uniform colloidal C-ZIF-8 particles with isotropic spherical PS particles, resulting in the formation of 2D binary superstructures (Figure 1a). For the spherical particles, we used aqueous colloids of sulfonated PS particles having a diameter of 600 nm and a surface charge of *ca.* −51 mV (Figure S1). Conversely, we synthesized C-ZIF-8 particles having two different edge sizes by adding an aqueous solution of Zn(NO_3_)_2_·6H_2_O into an aqueous mixture of 2-methylimidazole (2-MiM) and cetyltrimethylammonium bromide (CTAB). Importantly, we were able to control the particle size by adjusting the amount of 2-MiM. The resulting mixtures were left undisturbed for 24 h at room temperature, and the resulting C-ZIF-8 particles were harvested *via* centrifugation, washed with deionized water, and finally stored as wet pellets. Analysis *via* field-emission scanning electron microscopy (FESEM), powder X-ray diffraction (PXRD), and zeta-potential measurements of the resulting colloid confirmed the formation of homogeneous C-ZIF-8 particles having an edge size of either 135 ± 7 or 196 ± 12 nm and a surface charge of *ca.* +46 mV (Figures S1–S4).

**Figure 1:**
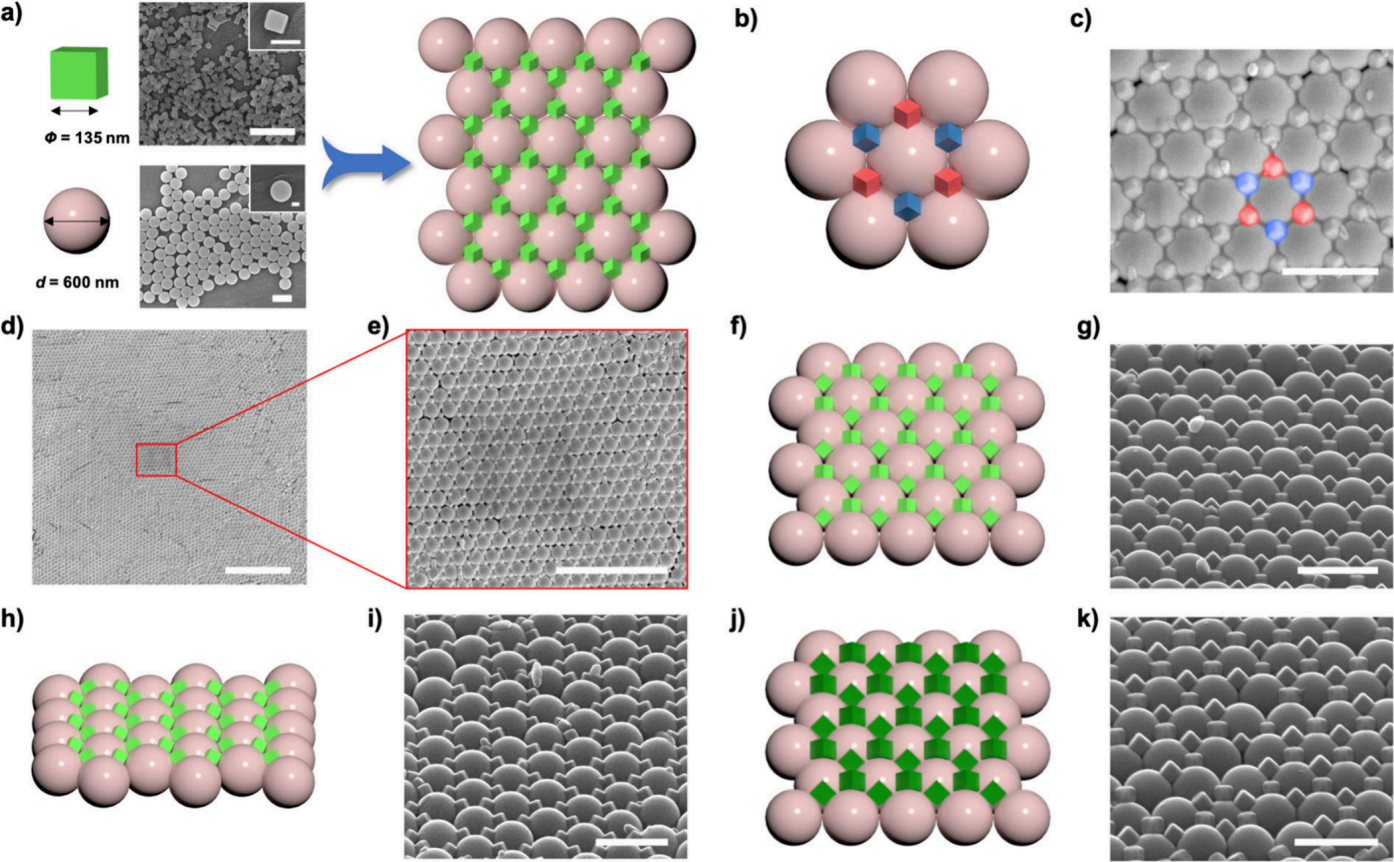
(a) Schematics and FESEM images of the coassembly of C-ZIF-8 particles and PS particles into 2D binary superstructures. (b, c) Schematic and FESEM images of the superstructure showing C-ZIF-8 particles assembled along two distinct orientations, highlighted in red and blue. (d, e) FESEM images of the superstructure under different magnifications. (f–i) Schematics and FESEM images of a 45°-tilted 2D binary superstructure viewed from different angles. (j, k) Schematic and FESEM image of a 45°-tilted 2D binary superstructure made of C-ZIF-8 particles (edge size: 196 ± 12 nm). Scale bars in (a), (c), (g), (i), (k): 1 μm. Scale bars in insets: (a, b) 200 nm; (d) 10 μm; (e) 5 μm.

Next, we attempted to coassemble the C-ZIF-8 and spherical PS particles into ordered binary superstructures by evaporation-induced self-assembly. Briefly, positively charged C-ZIF-8 particles and negatively charged PS particles were individually equilibrated in an aqueous solution containing 0.08 mM Pluronic F127 and the desired amount of NaCl. Following a 1 h equilibration period, the two suspensions were combined by vortexing and then further equilibrated for an additional 15 min. Upon completion of the equilibration process, 50 μL of the colloidal mixture was added to a pristine substrate, and the resulting droplet was allowed to dry overnight in an oven (40 °C). Examination *via* FESEM revealed the formation of a 2D ordered superstructure from the coassembly of C-ZIF-8 and PS particles (Figure 1). Importantly, the NaCl concentration and the evaporation conditions were crucial in regulating the ordering of the two particle types. Indeed, upon making slight alterations to the NaCl concentration (2–6 mM) or to the evaporation temperature (35 or 45 °C instead of 40 °C), we observed significant reduction in their ordering degree (Figures S5 and S6).

The FESEM images revealed the formation of a 2D binary superstructure wherein the PS spheres assembled in a hexagonal pattern and the C-ZIF-8 particles gathered in the triangular interstices created by three spherical PS particles within the hexagonal arrangement (Figure 1). Intriguingly, we observed that the C-ZIF-8 particles within this binary superstructure, despite sharing the same (111) orientation, exhibit two distinct orientations, highlighted in red and blue in Figure 1b,c. When spherical particles arrange themselves in a hexagonal pattern, they generate triangular interstices displaying two different orientations: one with the vertex of the “triangle” pointing upward (red cubes in Figures 1b,c) and one with it pointing downward (blue cubes in Figure 1b,c). As C-ZIF-8 particles are arranged within these interstices such that three faces of the cube contact the surfaces of three spherical PS particles, the varying orientations of the triangular interstices influence the assembly and orientation of the C-ZIF-8 particles (Figures 1f–k and S7).

To further investigate the coassembly of PS and C-ZIF-8 particles, we also examined whether this coassembly could be achieved using a preassembled 2D hexagonal arrangement of PS spheres as a template. To this end, we placed 15 μL of a colloidal solution of C-ZIF-8 particles at different concentrations onto a preassembled hexagonal arrangement, and the resulting droplets were allowed to dry in an oven (40 °C) overnight. The FESEM images showed that the C-ZIF-8 particles were not preferentially positioned in the triangular interstices of the hexagonal arrangement but rather randomly aggregated on the PS spheres (Figure S8). These results indicated that formation of the long-range-ordered binary superstructures would require coassembly of the two particle types in solution.

Having demonstrated that using oppositely charged particles in conjunction with PACS is an efficient strategy for coassembling polyhedral MOF particles with PS spheres, we next endeavored to replace the isotropic PS spheres with another anisotropic ZIF-8 particle. Here we aimed to enable the formation of a 3D binary superstructure *via* coassembly of two types of polyhedral ZIF-8 particles. We chose TRD-ZIF-8 particles, theorizing that they could coassemble with the C-ZIF-8 particles through their square facets. We envisioned that the shape complementarity could induce the formation of an fcc NaCl-type superstructure (Figure 2) in which each C-ZIF-8 particle would be surrounded by six other TRD-ZIF-8 particles and each TRD-ZIF-8 particle would be encompassed by six other C-ZIF-8 particles.

**Figure 2:**
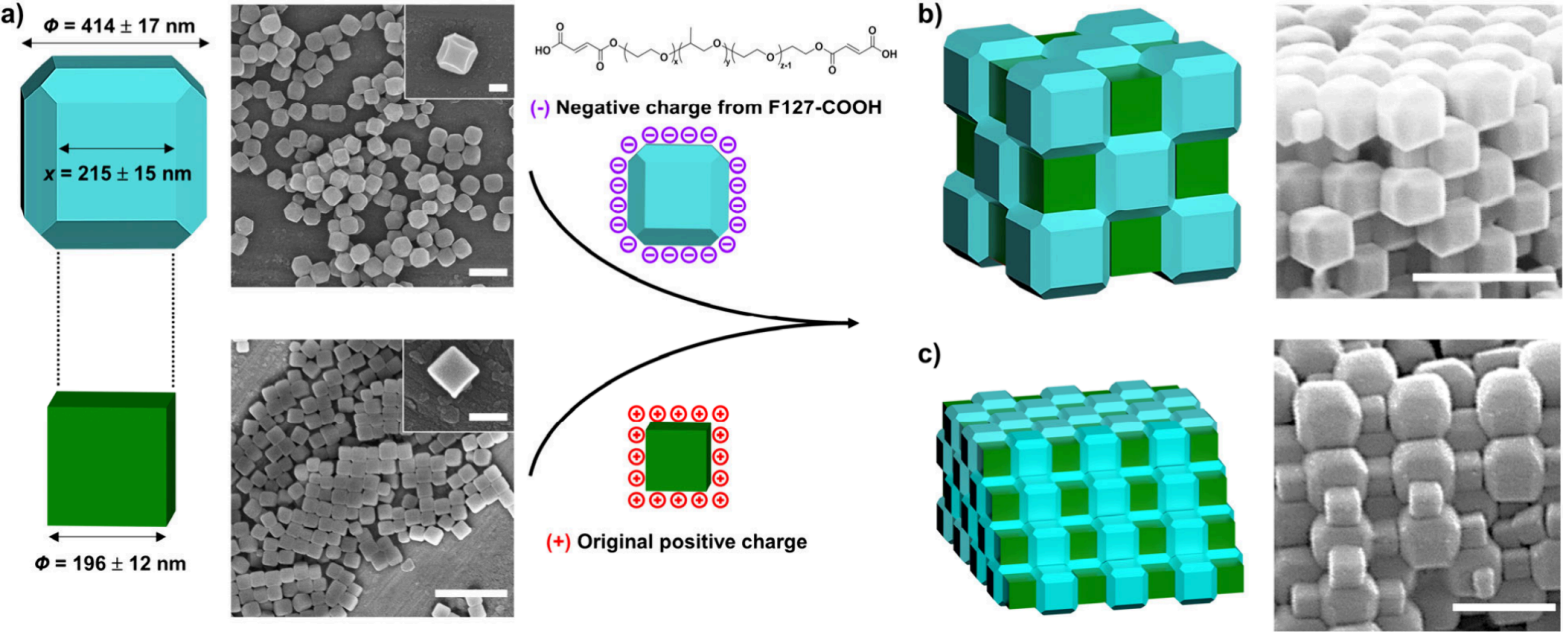
Schematics and FESEM images of (a) C-ZIF-8 and TRD-ZIF-8 particles and (b, c) the fcc NaCl-type porous binary superstructure, showing its 3D character. Scale bars: (a–c) 1 μm; (insets of a) 200 nm.

In addition to the shape complementarity between the two particle types, another necessary condition for the formation of our desired superstructures is size complementarity (Figure 2a). In our case, the edge size of the C-ZIF-8 particles had to be similar to the edge size of the truncated square face of the TRD-ZIF-8 particles. Accordingly, we selected positively charged C-ZIF-8 particles having an edge size of 196 ± 12 nm and synthesized TRD-ZIF-8 particles having an edge size (of the truncated square face) of 215 ± 15 nm (Figures 2a and S9). Synthesis of the TRD-ZIF-8 particles began with addition of an aqueous solution of Zn(OAc)_2_·2H_2_O to an aqueous solution of 2-MiM and CTAB. Next, the resulting mixture was gently stirred for 15 s, leading to the formation of a white colloidal suspension, which was then left undisturbed at room temperature for 2 h. The resulting TRD-ZIF-8 particles were then collected *via* centrifugation, washed with deionized water, and finally stored as wet pellets.

Another crucial condition for the formation of our desired superstructure is that the two types of polyhedral particles be oppositely charged. However, the synthesized C-ZIF-8 particles and TRD-ZIF-8 particles each exhibited a positive surface charge, ranging from +41 to +46 mV. To address this, we modified the surface charge of the TRD-ZIF-8 particles by functionalizing them with a presynthesized polymer, F127-COOH, containing terminal carboxylic acid groups (Figures 2a and S10). Briefly, 280 mg of TRD-ZIF-8 particles was dispersed in 20 mL of a 0.8 mM aqueous solution of F127-COOH with 20 mL of tetrahydrofuran (THF), and the resultant solution was allowed to equilibrate for 1 h. Note that using THF is important in this step because it enhances the attachment of F127-COOH onto the TRD-ZIF-8 particles (Figure S11). Zeta-potential measurements confirmed that the resultant F127-COOH-coated TRD-ZIF-8 particles exhibited a negative surface charge of *ca.* −22 mV (Figure S11). Next, the positively charged C-ZIF-8 particles and the negatively charged TRD-ZIF-8 particles were separately equilibrated in aqueous solutions containing 0.8 mM of Pluronic F127 and 3.0 mM NaCl. After 1 h, the two suspensions were mixed using a vortex mixer and then equilibrated for another 15 min. Following equilibration, 2.5 mL of the colloidal mixture was added into a cup lined with aluminum foil and then left to dry in an oven (40 °C) overnight.

FESEM images of the dried sample revealed the formation of 3D fcc NaCl-type ordered superstructures resulting from the coassembly of C-ZIF-8 and TRD-ZIF-8 particles (Figures 2 and 3). Schematic and FESEM images of the cross section illustrated the ordered arrangement of the two shape-complementary particles (Figure 2b,c). Upon coassembly, both types of ZIF-8 particles adopted the characteristic NaCl-type superstructure, wherein each C-ZIF-8 particle links, in 3D, six TRD-ZIF-8 particles through their square facets. With this arrangement, both types of ZIF-8 particles are aligned with their ⟨100⟩ directions perpendicular to the surface of the superstructure. PXRD of the superstructures confirmed this alignment, revealing the peak of greatest intensity at 2θ = 11°, corresponding to the diffraction from (002) planes of ZIF-8 particles (Figure 3h).^45,46^ This observation further confirmed the long-range ordering of the superstructures.

**Figure 3:**
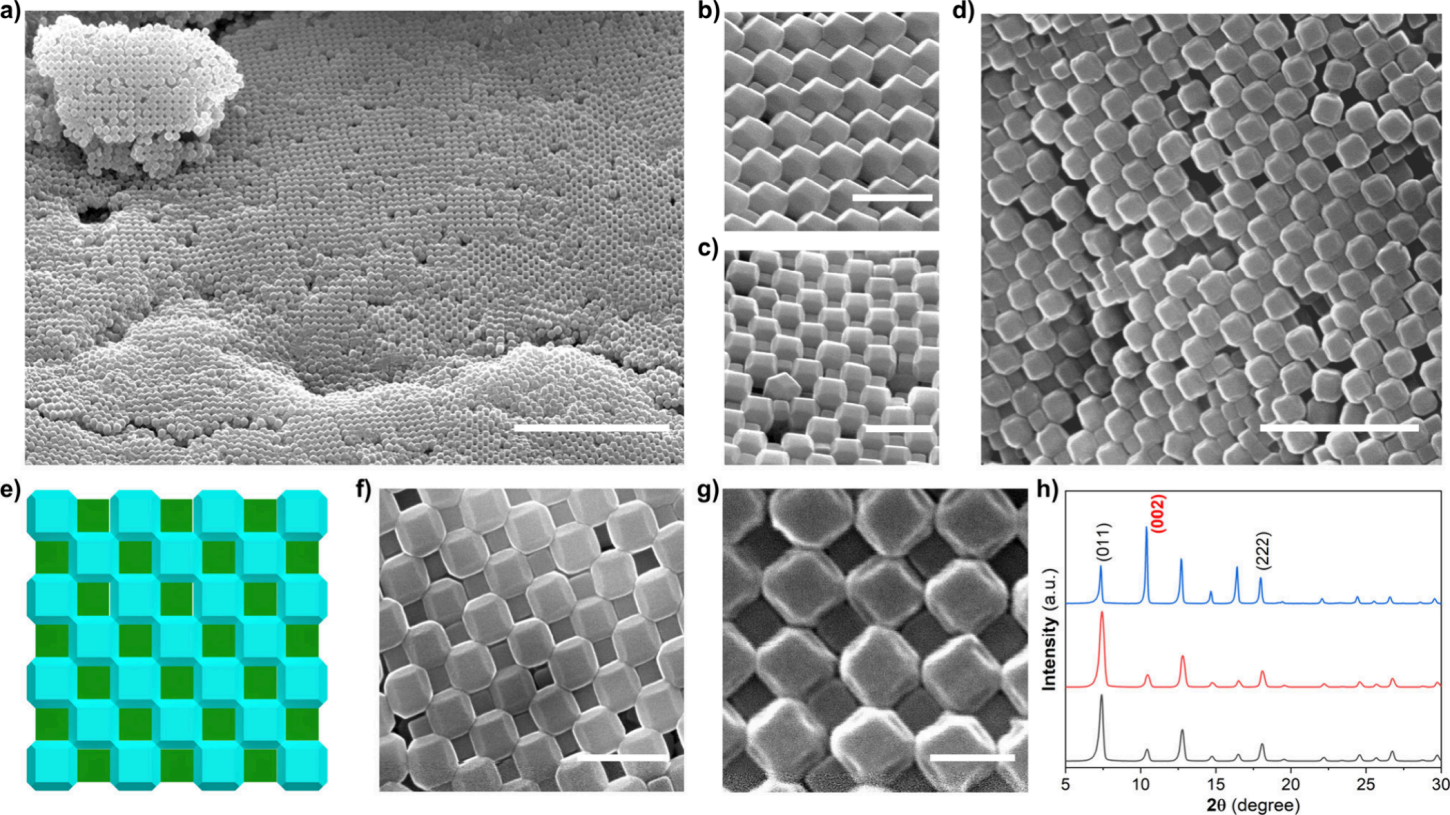
(a–c) FESEM images of a 45°-tilted NaCl-type porous binary superstructure showing (a) long-range order and (b, c) magnified views. (d–g) Schematic and FESEM images of top views of the NaCl-type porous binary superstructure. (h) PXRD patterns of disordered C-ZIF-8 particles (black), TRD-ZIF-8 particles (red), and the ordered binary superstructure (blue). Scale bars: (a) 10 μm; (d) 2 μm; (b,c,f) 1 μm; (g) 400 nm.

In conclusion, we have demonstrated that different types of polyhedral MOF particles can be coassembled into ordered binary porous superstructures, provided that the size, shape, and charge of the particles are complementary. We anticipate that this demonstration will benefit the self-assembly field by leveraging the versatility of MOF materials to fabricate sophisticated, ordered, self-assembled materials that comprise particles having various sizes, shapes, charges, porosities, or chemical compositions.

## Supporting Information Available

Detailed synthesis and methods, zeta-potential measurements, FESEM images, and PXRD diffractograms (PDF)
