# Supplementary material for: Coassembly of Complementary Polyhedral Metal–Organic Framework Particles into Binary Ordered Superstructures
Source: J Am Chem Soc. 2024 Jul 26;146(31):21225–30. doi: 10.1021/jacs.4c07194 (PMC11311218; doi:10.1021/jacs.4c07194)
Supplement: Supplementary file 1 — ja4c07194_si_001.pdf [file ja4c07194_si_001.pdf]

Co-assembly of Complementary Polyhedral Metal-Organic Framework Particles into Binary Ordered Superstructures

Lingxin Meng,[†] Javier Fonseca,[†] Roberto Sánchez-Naya,[†,‡] Amir Mohammad Ghadiri,[†] Inhar Imaz,[*,†,‡] and Daniel Maspoch[*,†,‡,§]

†Catalan Institute of Nanoscience and Nanotechnology (ICN2), CSIC, and Barcelona Institute of Science and Technology, 08193 Bellaterra, Barcelona, Spain.

‡Departament de Química, Facultat de Ciències, Universitat Autònoma de Barcelona, 08193 Bellaterra, Spain.

§ICREA, Pg. Lluís Companys 23, 08010 Barcelona, Spain.

inhar.imaz@icn2.cat

daniel.maspoch@icn2.cat

<u>Table of contents:</u>

1. Chemicals and instrumentation: ·· **4**

1.1. Chemicals 4

1.2. Instrumentation... 4

2. Materials and methods: ··· **5**

2.1. Synthesis of C-ZIF-8 particles (edge size = 135 ± 7 nm)... 5

2.2. Synthesis of C-ZIF-8 particles (edge size = 196 ± 12 nm)... 5

2.3. Synthesis of TRD-ZIF-8 particles (edge size = 414 ± 17 nm) 5

2.4. Synthesis and purification of Pluronic F127-COOH.. 5

2.5. Co-assembly of C-ZIF-8 (edge size = 135 ± 7 nm) and spherical PS particles 6

2.6. Co-assembly of C-ZIF-8 (edge size = 196 ± 12 nm) and spherical PS particles 6

2.7. Functionalization of TRD-ZIF-8 (edge size = 414 ± 17 nm) particles with F127-COOH.......... 6

2.8. Co-assembly of TRD-ZIF-8 (edge size = 414 ± 17 nm) and C-ZIF-8 (edge size = 196 ± 12 nm) particles... 6

3. Characterization ·· **8**

Figure S1. Zeta-potential measurements of PS and C-ZIF-8 colloidal particles............................... 8

Figure S2. Size-distribution of C-ZIF-8 particles (edge size = 135 ± 7 nm).................................... 9

Figure S3. Size-distribution of C-ZIF-8 particles (edge size = 196 ± 12 nm)................................. 10

Figure S4. Simulated and synthesized PXRD patterns of ZIF-8 particles 11

Figure S5. FESEM images of the co-assembly of C-ZIF-8 (edge size = 135 ± 7 nm) and 600 nm-in-diameter spherical PS particles at different NaCl concentrations (2-6 mM).................................. 12

Figure S6. FESEM images of the co-assembly of C-ZIF-8 (edge size = 135 ± 7 nm) and 600 nm-in-diameter spherical PS particles at different temperatures (35 - 70 °C). 13

Figure S7. FESEM images of the co-assembly of C-ZIF-8 (edge size = 196 ± 12 nm) and 600 nm-in-diameter spherical PS particles 14

Figure S8. FESEM images of the assembly of C-ZIF-8 particles (edge size = 135 ± 7 nm) on a pre-assembled 2D hexagonal arrangement of PS spheres. 15

Figure S9. Size-distribution of TRD-ZIF-8 (edge size = 414 ± 17 nm) particles 16

Figure S10. ^{1}H NMR (300 MHz, $CDCl_3$) spectra of F127-COOH. 17

Figure S11. Zeta-potential measurements of TRD-ZIF-8 (edge size = 414 ± 17 nm) colloidal particles 18

4. References 19

1. Chemicals and instrumentation:

1.1. Chemicals

Zinc acetate dihydrate ($Zn(CH_3COO)_2·2H_2O$, 98%), Cetyltrimethylammonium bromide (CTAB, 98%), Pluronic® F-127, maleic anhydride (99%), aluminum oxide 90 active basic and deuterochloroform ($CDCl_3$, 99.8 atom % D) were purchased from Sigma-Aldrich Co. 2-methylimidazole (2-MiM, 98%) was purchased from TCI Chemical. Zinc nitrate hexahydrate ($Zn(NO_3)_2·6H_2O$, 99%) was purchased from Alfa Aesar. 600 nm sulfate polystyrene (PS) particles, chloroform ($CHCl_3$, 99.8%), diethyl ether (Et_2O, 99%), tetrahydrofuran (THF, 99.5%) and dialysis cassettes (3.5K MWCO) were purchased from Thermo Fisher. All chemical reagents and solvents were used as received without further purification, unless otherwise specified. De-ionized (DI) water was obtained from a Milli-Q water purification system (18.2 MΩ·cm).

1.2. Instrumentation

Field-emission scanning electron microscopy (FESEM) images were collected on scanning electron microscopes (FEI Magellan 400L XHR and Quanta 650 FEG), using conductive aluminum or copper tape. The sizes of crystals were calculated from FESEM images by averaging the distance of at least 200 particles from images of different areas of the same samples. Particles vortexing and equilibration were conducted by IKA VORTEX 3 and IKA Trayster digital. Powder X-ray diffraction (PXRD) measurements were made on an X'Pert PRO MPD diffractometer (Panalytical), with $\lambda Cu = 1.5406$ Å. The surface charge of particles expressed as zeta-potential (ζ) was measured using a Malvern Zetasizer, (Malvern Instruments, UK). Proton Nuclear Magnetic Resonance (^1H NMR) spectra were recorded on a Bruker Avance 300 MHz spectrometer.

2. Materials and methods:

2.1. Synthesis of C-ZIF-8 particles (edge size = 135 ± 7 nm)

C-ZIF-8 (edge size = 135 ± 7 nm) was synthesized adapted from a previously described methodology.[1] In a typical synthesis, 24 mL of an aqueous solution containing 2-MiM (2880 mg) was mixed with 4 mL of 1 mg/mL CTAB aqueous solution. Then, an aqueous solution (8 mL) containing $Zn(NO_3)_2{\cdot}6H_2O$ (140 mg) was added into the previous mixture. After standing for 24 h, the resulting ZIF-8 particles were washed with DI water (10 mL) upon centrifugation at 9000 rpm in 50-mL Falcon tubes. ZIF-8 particles (~100 mg) were finally stored as wet pellets.

2.2. Synthesis of C-ZIF-8 particles (edge size = 196 ± 12 nm)

C-ZIF-8 (edge size = 196 ± 12 nm) was synthesized adapted from a previously described methodology.[1] In a typical synthesis, 24 mL of an aqueous solution containing 2-MiM (1920 mg) was mixed with 4 mL of 1 mg/mL CTAB aqueous solution. Then, an aqueous solution (8 mL) containing $Zn(NO_3)_2{\cdot}6H_2O$ (140 mg) was added into the previous mixture. After standing for 24 h, the resulting ZIF-8 particles were washed with DI water (10 mL) upon centrifugation at 9000 rpm in 50-mL Falcon tubes. ZIF-8 particles (~100 mg) were finally stored as wet pellets.

2.3. Synthesis of TRD-ZIF-8 particles (edge size = 414 ± 17 nm)

TRD-ZIF-8 (edge size = 414 ± 17 nm) was synthesized adapted from a previously described methodology.[2] In a typical synthesis, a solution of $Zn(CH_3COO)_2{\cdot}2H_2O$ (300 mg) in 5 mL of DI water was added into a solution of 1.12 g of 2-MiM and 0.40 mg CTAB in 5 mL of DI water. The resulting mixture was gently stirred for 15 s, causing it to evolve into a white colloidal suspension, which was left undisturbed at room temperature for 2 h. The resulting ZIF-8 particles were washed three times with deionized (DI) water upon centrifugation at 9000 rpm in 50 mL Falcon tubes. ZIF-8 particles (~280 mg) were finally stored as wet pellets.

2.4. Synthesis and purification of Pluronic F127-COOH

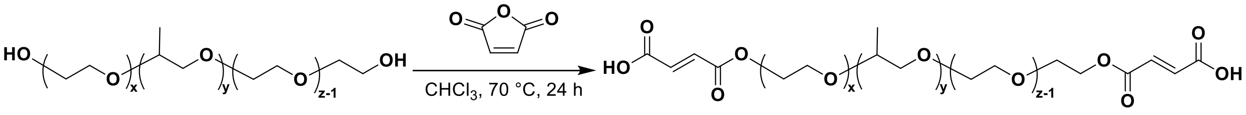

Pluronic F127-COOH was synthesized according to the previously described methodology.[3] $CHCl_3$ was first passed through an activated basic aluminum oxide column to remove the stabilizer EtOH from commercial $CHCl_3$. Then, F127 (20 g, 1.6 mmol) and maleic anhydride (3.4 g, 35 mmol) were dissolved in

$CHCl_3$ (100 mL), and the resulting solution was allowed to react for 24 h under stirring at 70 °C under nitrogen atmosphere. Following completion of the reaction, the solution was concentrated and poured three times into an excess amount of ice-cold Et_2O to precipitate the reaction product. After precipitation, the product was dried under reduced pressure, dissolved in water to prepare a 10% (w/v) solution and dialyzed (3.5K MWCO) against 1000 mL DI water for 6 days. The DI water was exchanged twice every day during dialysis. Finally, F127-COOH was dried by lyophilization and collected as a white power. Note that dialysis is vital to remove the electrolyte impurities emerged during the synthesis (*e.g.*, maleic acid), as the electrolytes seriously affect the functionalization process of the TRD-ZIF-8 particles.

2.5. Co-assembly of C-ZIF-8 (edge size = 135 ± 7 nm) and spherical PS particles

Positively charged C-ZIF-8 (10 mg/mL) and negatively charged PS particles (82 mg/mL) were separately equilibrated in an aqueous solution containing 0.08 mM of Pluronic F127 and 3.0 mM of NaCl. After 1 h, the two suspensions were mixed by vortexing and then equilibrated for another 15 min. After equilibration, 50 μL of the colloidal mixture was added dropwise onto a clean glass slide and the treated sample was left to dry in an oven at 40 ˚C overnight.

2.6. Co-assembly of C-ZIF-8 (edge size = 196 ± 12 nm) and spherical PS particles

Positively charged C-ZIF-8 (10 mg/mL) and negatively charged PS particles (82 mg/mL) were separately equilibrated in an aqueous solution containing 0.08 mM of Pluronic F127 and 3.5 mM of NaCl. After 1 h, the two suspensions were mixed by vortexing and then equilibrated for another 15 min. After equilibration, 50 μL of the colloidal mixture was added dropwise onto a clean glass slide and the treated sample was left to dry in an oven at 40 ˚C overnight.

2.7. Functionalization of TRD-ZIF-8 (edge size = 414 ± 17 nm) particles with F127-COOH

280 mg of TRD-ZIF-8 were dispersed in a 20 mL aqueous solution of 0.8 mM F127-COOH and 20 mL of THF, and the resulting dispersion was equilibrated for 1 h. Note here that using THF in this step is important because it enhances the attachment of F127-COOH on the TRD-ZIF-8 particles. After coating, the ZIF-8 particles were washed twice and finally dispersed in a 10 mL aqueous solution containing 0.8 mM of Pluronic F127 and 3.0 mM of NaCl at a final concentration of ZIF-8 particles of 28 mg/mL.

2.8. Co-assembly of TRD-ZIF-8 (edge size = 414 ± 17 nm) and C-ZIF-8 (edge size = 196 ± 12 nm) particles

Positively charged C-ZIF-8 (10 mg/mL) and F127-COOH coated negatively charged TRD-ZIF-8 particles (28 mg/mL) were separately equilibrated in aqueous solutions containing 0.8 mM of Pluronic F127 and 3.0 mM of NaCl. After 1 h, the two suspensions were mixed by vortex mixer and then equilibrated for another

15 min. After equilibration, 2.5 mL of the colloidal mixture were added onto a homemade aluminum foil liner cup and left to dry in an oven at 40 ˚C overnight.

3. Characterization

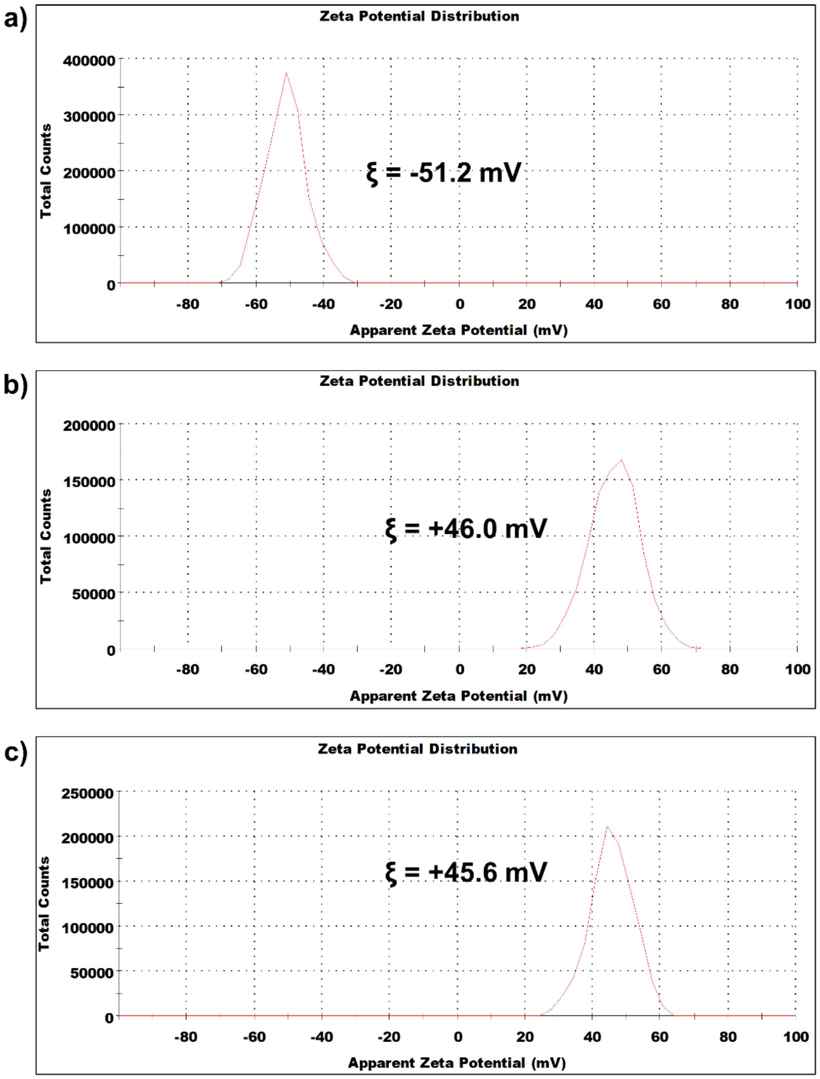

Figure S1. Zeta-potential measurements of PS and C-ZIF-8 colloidal particles: (a) 600 nm PS; (b) C-ZIF-8 (edge size = 135 ± 7 nm); and (c) C-ZIF-8 (edge size = 196 ± 12 nm).

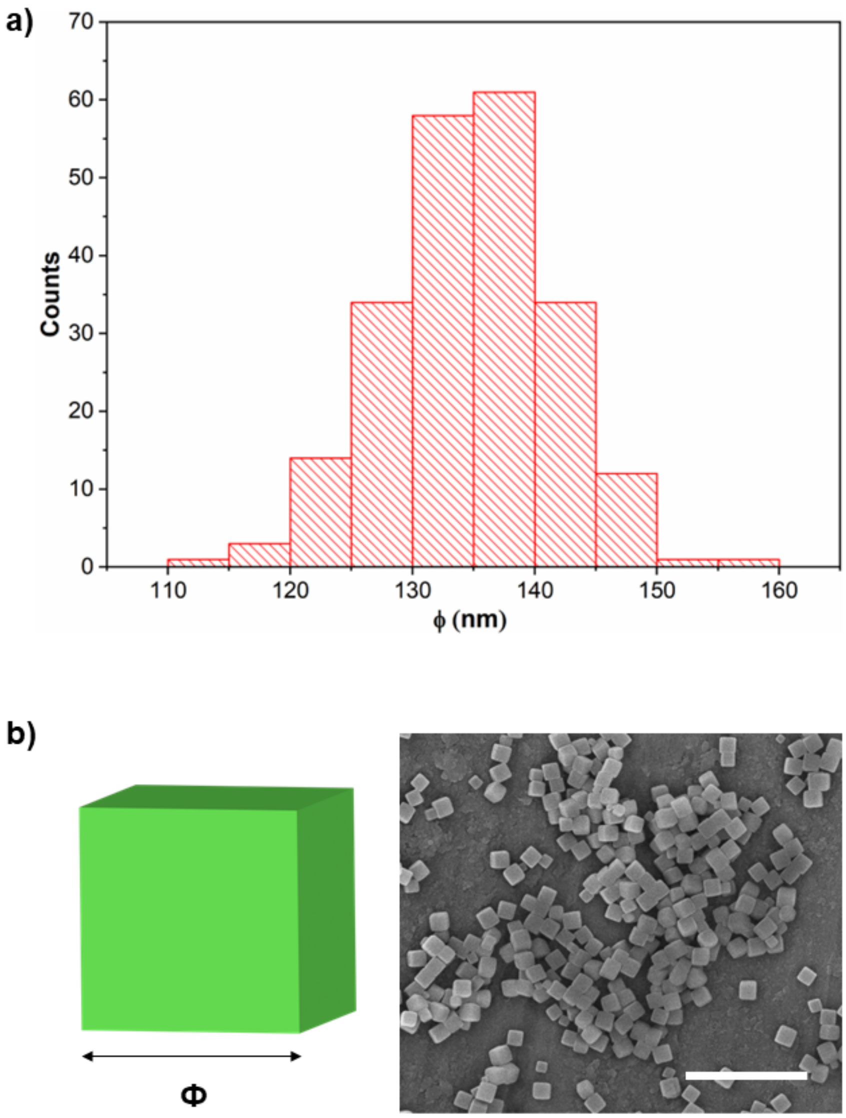

Figure S2. (a) Size-distribution histogram of C-ZIF-8 particles with an edge length ϕ of 135 ± 7 nm. (b) Scheme and FESEM image of C-ZIF-8 particles, highlighting the edge length of particles (ϕ). Scale bar: 1 μm.

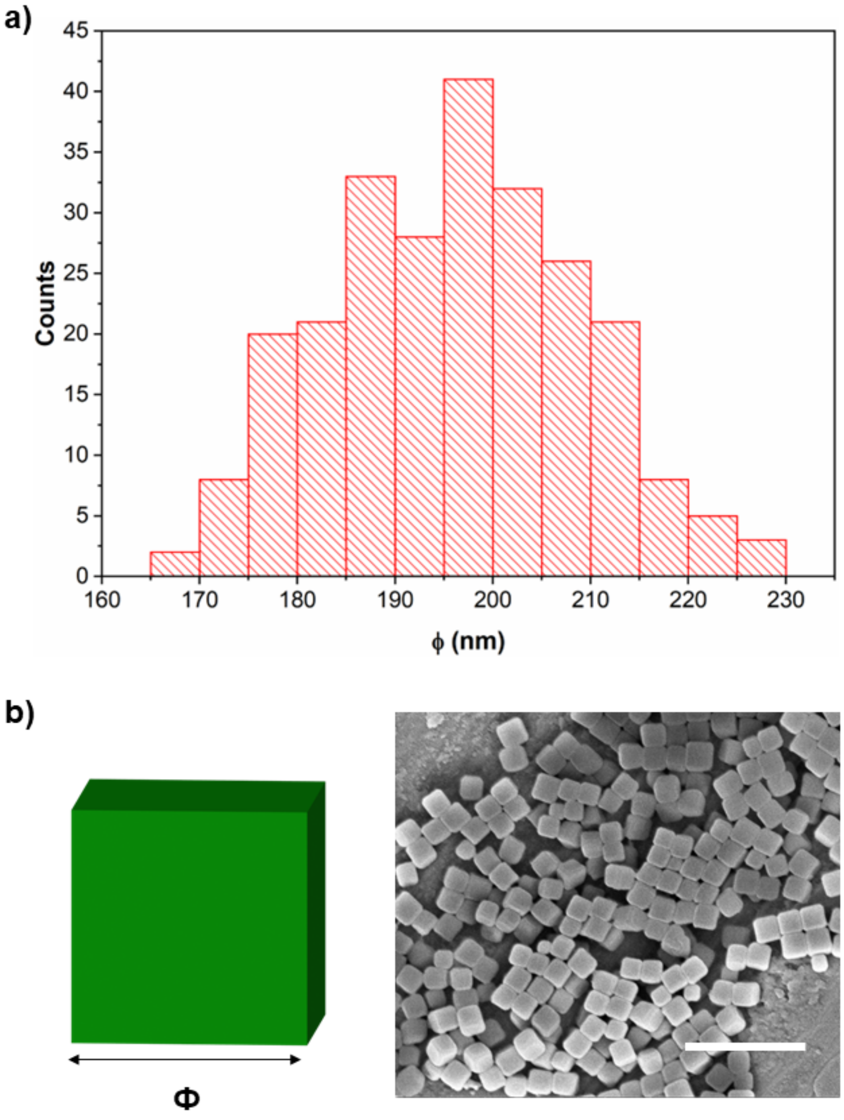

Figure S3. (a) Size-distribution histogram of C-ZIF-8 particles with an edge length ϕ of 196 ± 12 nm. (b) Scheme and FESEM image of C-ZIF-8 particles, highlighting the edge length of particles (ϕ). Scale bar: 1 μm.

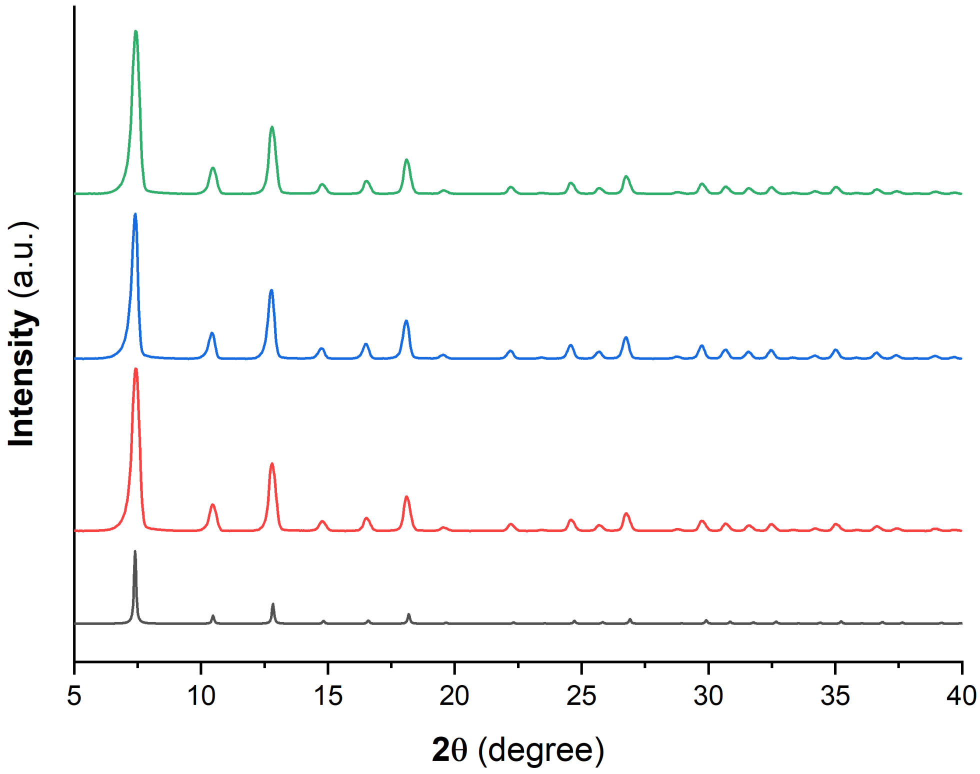

Figure S4. Simulated and synthesized PXRD patterns of ZIF-8 particles**:** C-ZIF-8 (red, edge size = 135 ± 7 nm); C-ZIF-8 (blue, edge size = 196 ± 12 nm); TRD-ZIF-8 (green, edge size = 414 ± 17 nm); and simulated ZIF-8 (black).

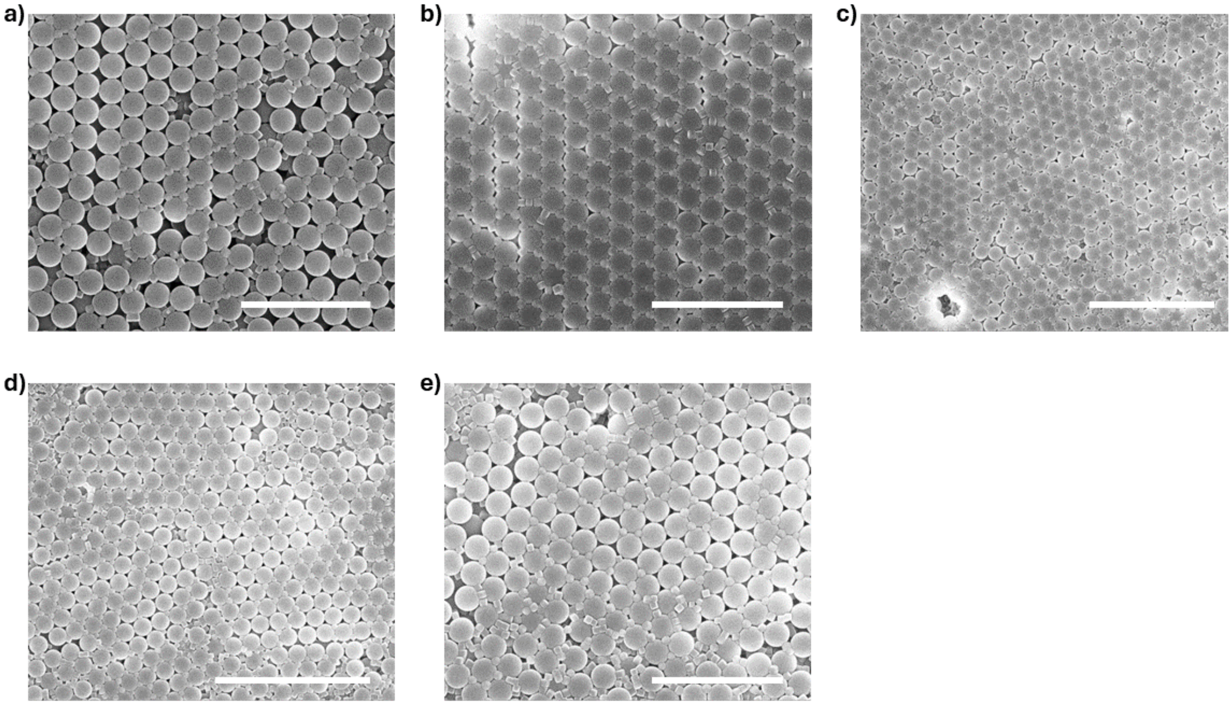

Figure S5. FESEM images of the co-assembly of C-ZIF-8 (edge size = 135 ± 7 nm) and 600 nm-in-diameter spherical PS particles at different NaCl concentrations (2-6 mM). The co-assembly experiments were performed according to the procedure described in Section 2.5, except that the concentration of NaCl was varied from: (a) 2.0 mM; (b) 3.0 mM; (c) 4.0 mM; (d) 5.0 mM; and (e) 6.0 mM. Note here that a concentration of 3.0 mM was chosen as the optimized concentration. Scale bars: 3 µm (a,b,e), and 5 µm (c,d).

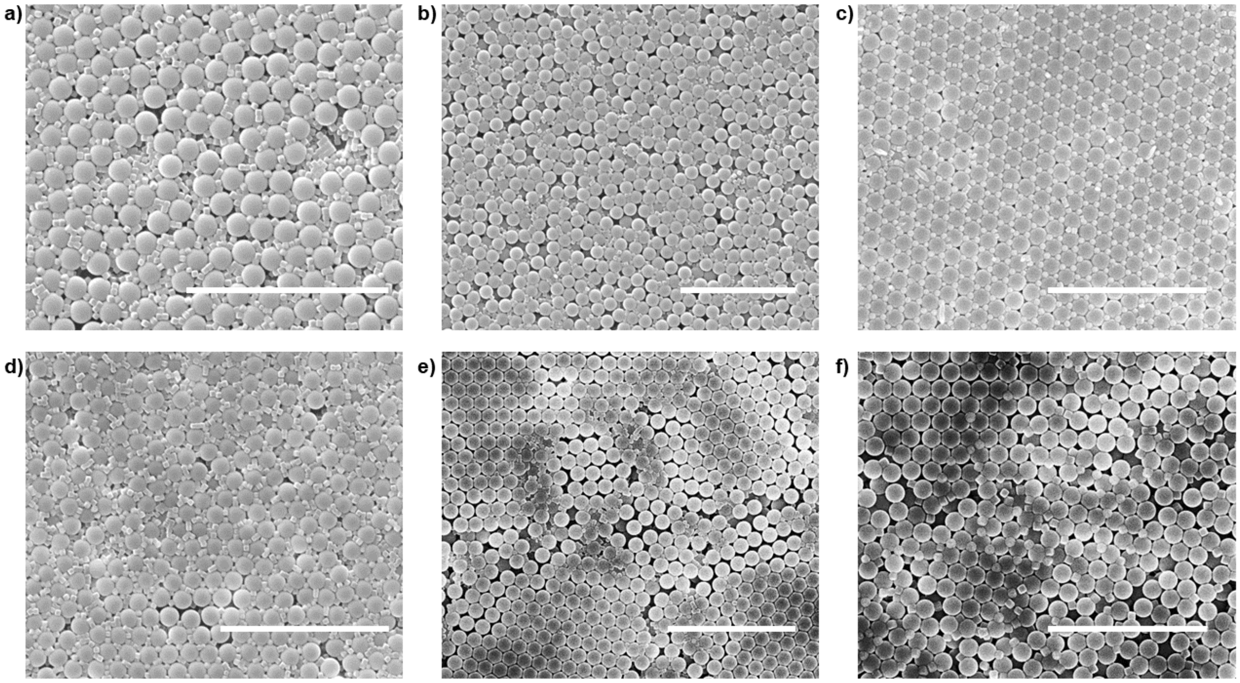

Figure S6. FESEM images of the co-assembly of C-ZIF-8 (edge size = 135 ± 7 nm) and 600 nm-in-diameter spherical PS particles at different temperatures (35 - 70 °C). The co-assembly experiments were performed according to the procedure described in Section 2.5, except that the drying temperature was varied from: (a) room temperature; (b) 35 °C; (c) 40 °C; (d) 45 °C; (e) 60 °C; and (f) 70 °C. Note that a temperature of 40 °C was chosen as the optimized temperature. Scale bars: 5 μm (a-f).

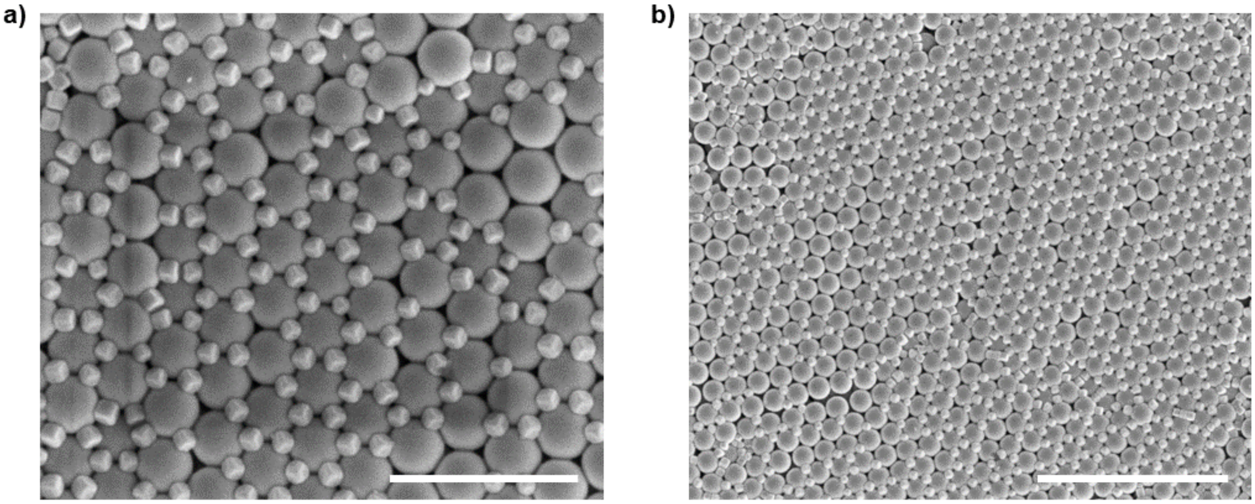

Figure S7. FESEM images of the co-assembly of C-ZIF-8 (edge size = 196 ± 12 nm) and 600 nm-in-diameter spherical PS particles. Scale bars: 2 µm (a); and 5 µm (b).

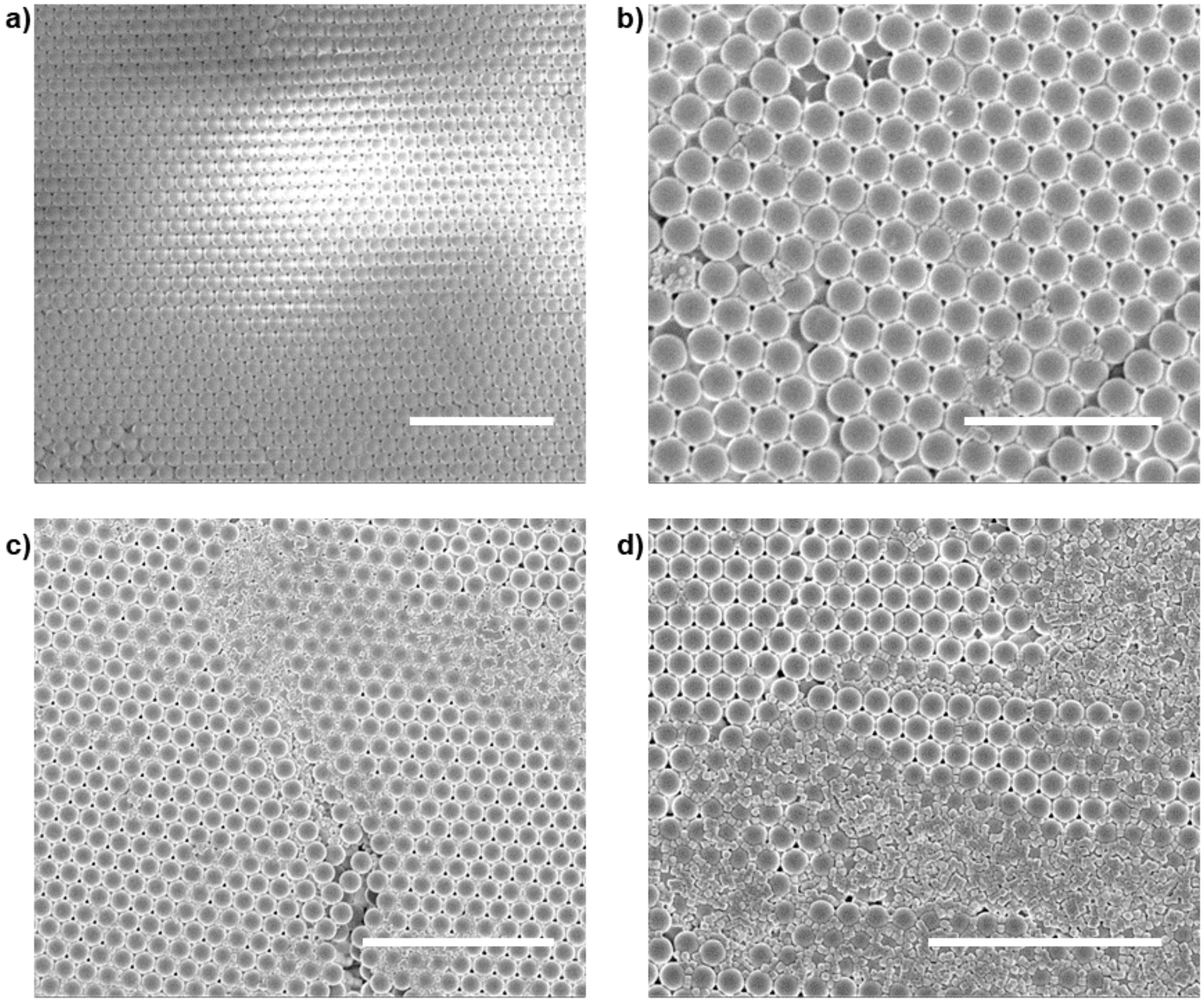

Figure S8. FESEM images of the assembly of C-ZIF-8 particles (edge size = 135 ± 7 nm) on a pre-assembled 2D hexagonal arrangement of PS spheres. (a) FESEM images of the pre-assembled 2D hexagonal arrangement of PS spheres. To assemble these spheres, PS particles (82 mg/mL) were dispersed in an aqueous solution containing 0.08 mM F127 and 3 mM NaCl. Then, 25 µL of this colloid were dropped on the glass substrate and dried at 40 °C overnight. (b-d) FESEM images of C-ZIF-8 particles (edge size = 135 ± 7 nm) placed and dried on these hexagonal arrangements of PS spheres. To perform this experiment, C-ZIF-8 particles at different concentrations [0.1 mg/mL (b); 0.5 mg/mL (c); and 1 mg/mL (d)] were dispersed in an aqueous solution containing 0.08 mM F127 and 3 mM NaCl. Then, 15 µL of these colloids were dropped on the PS template, followed by drying at 40 °C overnight. Scale bars: 5 µm (a-d).

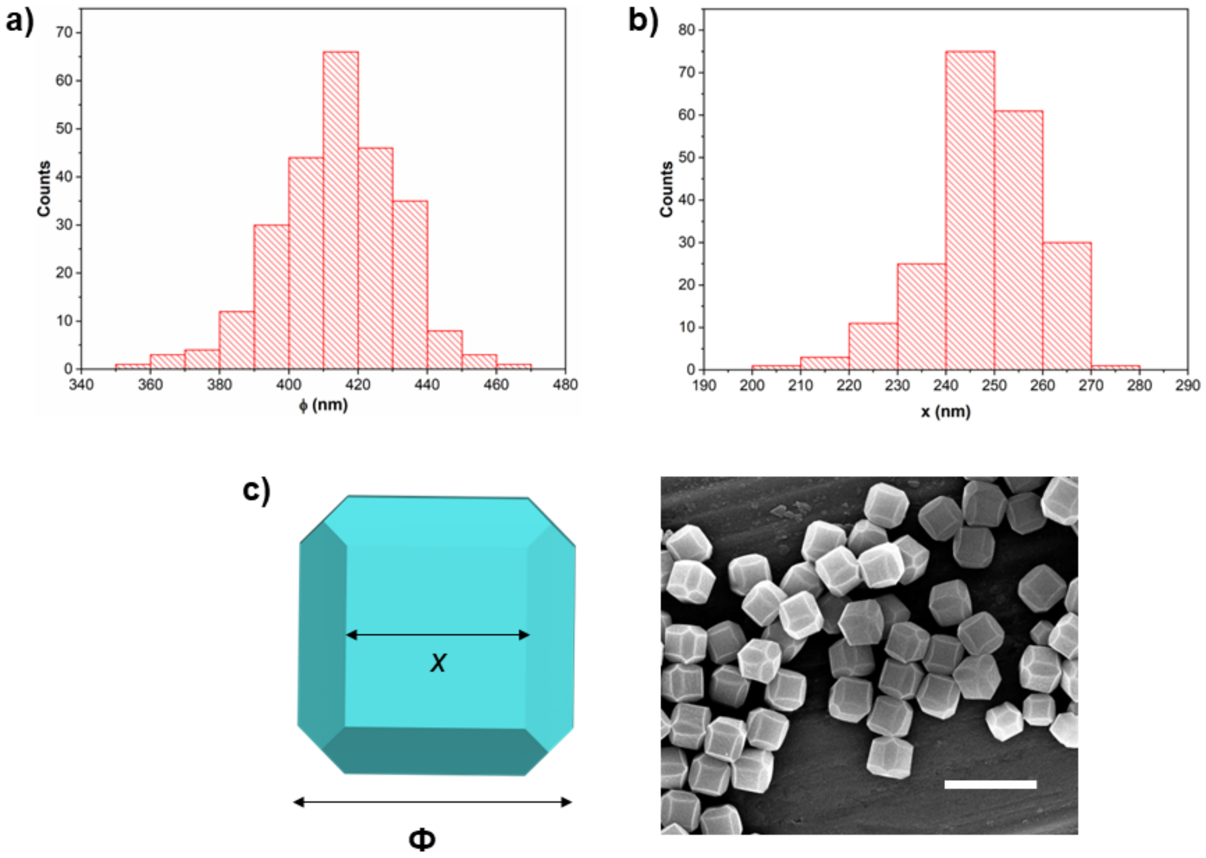

Figure S9. (a) Size-distribution histogram of TRD-ZIF-8 particles with an edge length ϕ of 414 ± 17 nm. (b) Size-distribution histogram of the edge of truncated square face of TRD-ZIF-8 with a square face edge length x of 215 ± 15 nm. (c) Scheme and FESEM image of TRD-ZIF-8 particles, highlighting the edge length of particles (ϕ) and the edge length of the truncated square face (x). Scale bar: 1 µm.

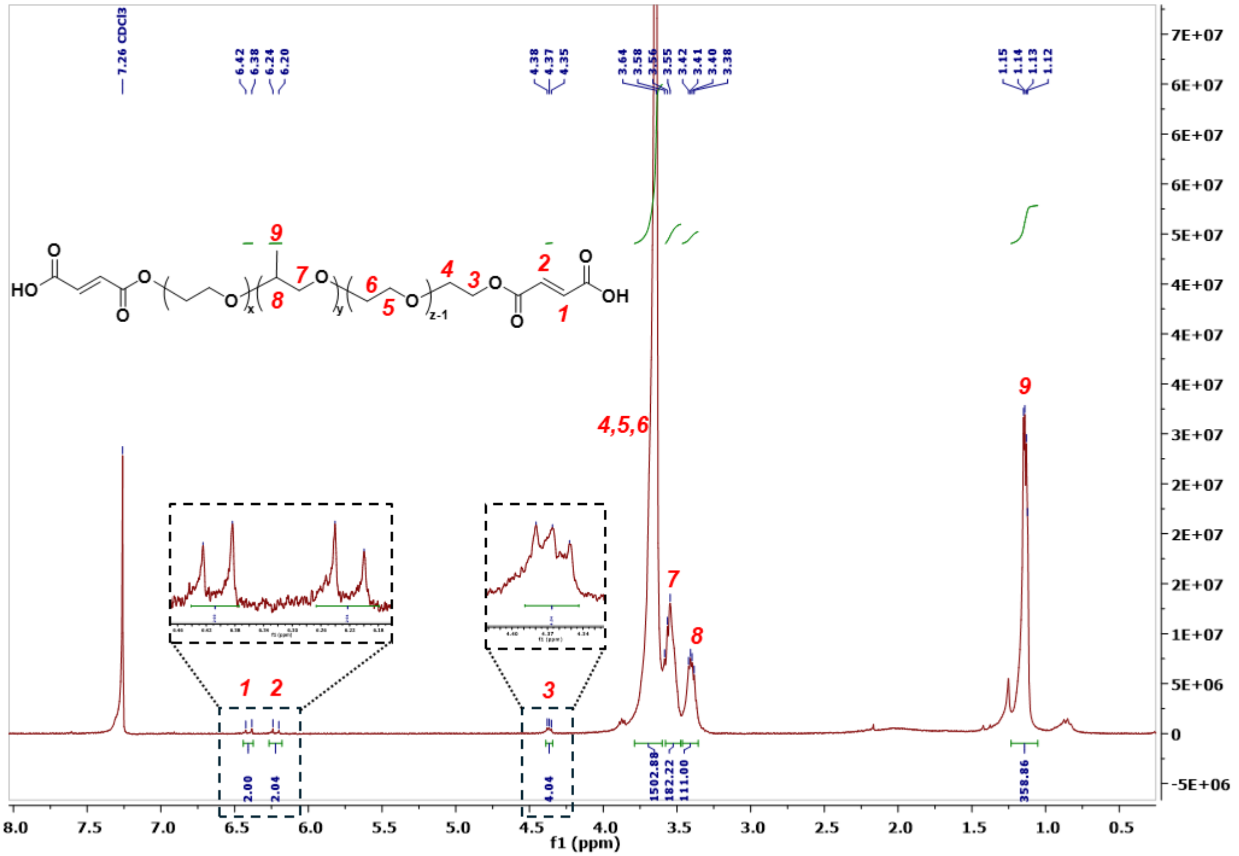

Figure S10. ^{1}H NMR (300 MHz, $CDCl_3$) spectra of F127-COOH. The presence of peaks at around 6.22 and 6.40 ppm are ascribed to protons **1** and **2** in F127-COOH, which indicates the conversion of the terminal hydroxyl groups of the F127 chains into carboxyl groups by reaction with maleic anhydride.

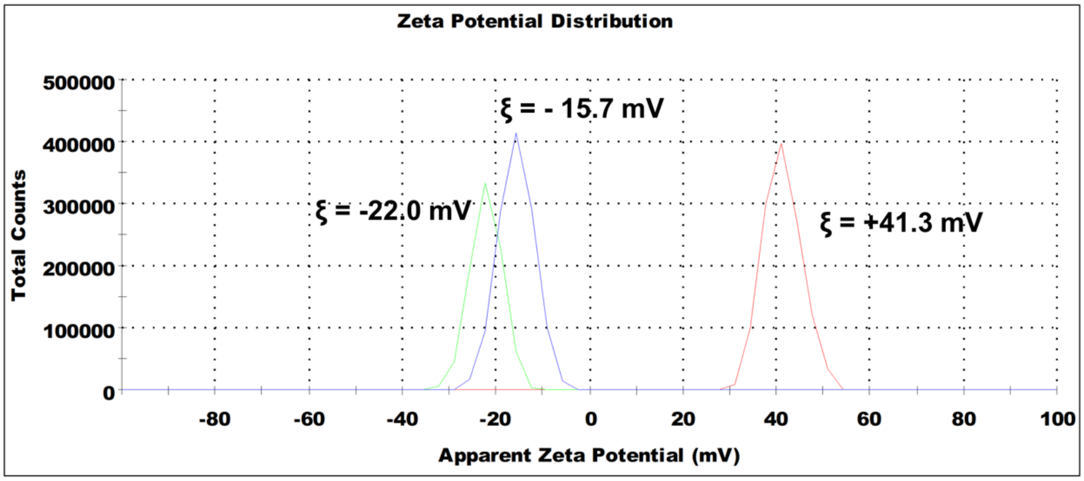

Figure S11. Zeta-potential measurements of TRD-ZIF-8 (edge size = 414 ± 17 nm) colloidal particles: TRD-ZIF-8 particles before (**red curve**) and after F127-COOH functionalization with THF (**green curve**). When the functionalization with F127-COOH was done without THF, the zeta potential value of the resulting TRD-ZIF-8 particles was less negative (**blue curve**) in comparison to that obtained when THF was used.

4. References

(1) Liu, Y.; Wang, J.; Imaz, I.; Maspoch, D. Assembly of Colloidal Clusters Driven by the Polyhedral Shape of Metal-Organic Framework Particles. *J. Am. Chem. Soc.* **2021**, *143* (33), 12943–12947.

(2) Avci, C.; Liu, Y.; Pariente, J. A.; Blanco, A.; Lopez, C.; Imaz, I.; Maspoch, D. Template-Free, Surfactant-Mediated Orientation of Self-Assembled Supercrystals of Metal–Organic Framework Particles. *Small* **2019**, *15* (31), 1902520.

(3) Li, Y.-Y.; Li, L.; Dong, H.-Q.; Cai, X.-J.; Ren, T.-B. Pluronic F127 Nanomicelles Engineered with Nuclear Localized Functionality for Targeted Drug Delivery. *Mater. Sci. Eng. C* **2013**, *33* (5), 2698–2707.
